# Morphological characterization, histopathological alteration, and cytokine response of different tissues of *Columba livia* naturally infected with *Haemoproteus columbae*

**DOI:** 10.3389/fvets.2025.1610416

**Published:** 2025-09-17

**Authors:** Heba M. Salem, Amira M. Ibrahim, Sara S. Barsoum, Mahmoud A. Mahmoud, Haleema H. Albohiri, Mina A. Almayouf, Layla A. Almutairi, Mohammed A. Alqahtani, Sultan Mohammed Areshi, Khaled A. El-Tarabily, Marwa M. Attia

**Affiliations:** ^1^Department of Poultry Diseases, Faculty of Veterinary Medicine, Cairo University, Giza, Egypt; ^2^Department of Poultry Diseases, Animal Health Research Institute, Giza, Egypt; ^3^Department of Zoology, Faculty of Science, Fayoum University, Fayoum, Egypt; ^4^Department of Pathology, Faculty of Veterinary Medicine, Cairo University, Giza, Egypt; ^5^Department of Biological Sciences, College of Science, University of Jeddah, Jeddah, Saudi Arabia; ^6^Department of Biology, College of Science, Qassim University, Buraydah, Saudi Arabia; ^7^Department of Biology, College of Science, Princess Nourah bint Abdulrahman University, Riyadh, Saudi Arabia; ^8^Department of Biology, College of Science, King Khalid University, Abha, Saudi Arabia; ^9^Department of Biology, College of Science, Jazan University, Jazan, Saudi Arabia; ^10^Department of Biology, College of Science, United Arab Emirates University, Al Ain, United Arab Emirates; ^11^Department of Parasitology, Faculty of Veterinary Medicine, Cairo University, Giza, Egypt

**Keywords:** blood parasites, cytochrome *b* gene, cytokine, gene expression, *Haemoproteus columbae*

## Abstract

**Introduction:**

*Haemoproteus columbae* is a common haemosporidian worldwide blood parasite affecting domestic pigeons (*Columba livia*). Therefore, this study aimed to detect the incidence of *H. columbae* infection in domestic pigeons with morpho-molecular identification.

**Methods:**

In the current study, blood samples were collected from 125 domestic pigeons between 2023 and 2024 and analyzed using both microscopic and molecular techniques. *H. columbae* positive birds underwent postmortem (PM) and histopathological examinations, as well as cytokine immunological reaction assessments.

**Results:**

It was found that around 8% (10/125) of pigeons were positive for *H. columbae* infection, and their morphological characteristics were reported. *H. columbae* induces observable macroscopic and microscopic alterations in the infected tissues, which increases the cytokine immunological reaction in the infected birds. The infected birds suffered from severe histopathological changes in most haemopoietic and parenchymatous organs. The transcript levels of inflammatory markers such as IL-6, IFN-γ, and IL-1β were significantly upregulated in *H. columbae*-infected birds. Additionally, the *H. columbae* samples’ mRNA level of the apoptotic Cas-3 indicated apoptotic activity.

**Discussion:**

Hematic parasites can pose a serious health threat to pigeons as they invade red blood cells and internal organs, leading to anemia, weakness, weight loss, and even death in severe cases. Epidemiological studies and surveys are essential for monitoring these hematologic parasites. Furthermore, additional research is recommended to evaluate the efficacy of various herbal extracts in comparison to the most frequently used drugs for managing this issue in affected pigeons.

## Introduction

Parasitic infections in poultry present a substantial economic burden due to their impact on bird health and productivity (1–3). Among these, avian haemosporidian parasites, particularly those from the genera *Plasmodium* and *Haemoproteus* (Apicomplexa: Haemosporidia), are widespread pathogens affecting both domestic and wild bird populations (4, 5).

One of the most studied avian haemosporidians is *Haemoproteus columbae*, a protozoan parasite primarily infecting pigeon (*Columba livia*) (6, 7). *H. columbae* follows a heteroxenous life cycle, with asexual replication occurring in erythrocytes and tissues of the avian host, and sexual reproduction taking place in blood-sucking dipterans, specifically louse flies (*Pseudolynchia canariensis*) (8, 9). Sporogony in the vector takes approximately 6.5–10 days (9).

Haemosporidians have traditionally been grouped into four genera: *Plasmodium*, *Haemoproteus*, *Leucocytozoon*, and *Fallisia* (10). The genus *Haemoproteus* is further divided into subgenera: *Haemoproteus* (transmitted by Hippoboscidae) and *Parahaemoproteus* (transmitted by Ceratopogonidae) based on vector specificity (11, 12).

Although often considered non-pathogenic in their natural avian hosts, *H. columbae* can cause significant pathology in immunologically naïve or stressed birds, especially in captivity. The parasite has been implicated in a disease commonly referred to as “pigeon malaria,” which can be fatal in young birds (13). Gametocytes characteristically appear in erythrocytes as crescent or horseshoe-shaped forms (13). Acute infections are often linked to pre-erythrocytic tissue stages, such as the rupture of megalomeronts in the liver and muscle, which can lead to systemic damage and mortality (14, 15).

Despite the global presence of avian haemosporidians, detailed studies focusing on the immune response and pathological alterations associated with natural *H. columbae* infections in pigeons remain scarce. In particular, there is a paucity of data integrating morphological, histopathological, and molecular findings with cytokine profiling to understand the host-pathogen interactions and potential consequences of infection.

Recently, it has been posited that pigeon haemoproteosis caused by *H. columbae* is predominantly found in tropical and subtropical regions. Therefore, the objective of this study is to identify the causative agents of mortality in pigeons, ascertain the incidence of *H. columbae* through morphological identification, and evaluate the tissue responses to this infection, including histopathological alterations and cytokine responses in various tissues of the infected pigeons.

## Materials and methods

### Ethical approval and consent to participate

The work was carried out according to the IACUC guidelines and was approved by the Ethical Committee, Faculty of Veterinary Medicine, Cairo University, Cairo, Egypt, with code “Vet CU 8032022407.”

### Collection of pigeons

For this study, 125 domestic pigeons of different ages (4 months to 4 years old) and mixed sex were collected from poultry markets and different poultry clinics. From the suspected birds with general signs of loss of weight, ruffled feathers, and general depression, blood samples were ethically collected from the wing vein.

In addition, apparently healthy birds confirmed to be negative for blood and gastrointestinal parasites via parasitological examination were kept as negative control birds.

### Parasitological examination

Blood samples were ethically collected from the wing vein to prepare blood smears. For every blood sample, two to three thin blood films were prepared on sterile glass slides, allowed to air-dry, and subsequently fixed in 100% methanol for 10 min. Giemsa’s solution (Sigma-Aldrich Chemie GmbH, Taufkirchen, Germany) was used to stain blood films.

The slides were examined under a 40× and 100× magnification (16) using Olympus BH-2 (Olympus Optical Co. Ltd., Tokyo, Japan) light microscope equipped with a digital camera and software (Jenoptik ProgRes Camera, C12plus, Frankfurt, Germany). The infection intensity was calculated using the number of infected erythrocytes (presence of male or female gametocytes) per 10 microscopic fields (17, 18).

The morphometric properties of the infected erythroparasites were microscopically assessed, and the morphological characteristics key established by Valkiūnas and Iezhova (19) was used to identify *H. columbae*.

### Transcript levels analysis of the infected spleen and lung with *H. columbae*

Lung and spleen tissues were aseptically collected from 10 *H. columbae*-infected pigeons and 10 uninfected controls. A tissue sample of 100 mg from each organ was homogenized using a FastPrep-24 system (MP Biomedicals, Irvine, California, United States) with two 30-s cycles at 6 m/s and then placed in Lysing Matrix D tubes. Total RNA was extracted using Ambion RNA isolation kits (Thermo Fisher Scientific Inc., Waltham, Massachusetts, United States), and its concentration and purity were assessed with a NanoDrop spectrophotometer (Thermo Fisher Scientific).

Subsequently, 500 ng of RNA per sample was treated with DNase I (Thermo Fisher Scientific) and converted to cDNA using the High-Capacity cDNA Archive Kit (Thermo Fisher Scientific). Quantitative real-time PCR was conducted using the Applied Biosystems 7500 platform with SYBR Green-based chemistry (Wiz Pure^™^ qPCR Master Mix, Wizbiosolutions Inc., Seongnam-si, South Korea) and ROX as the reference dye.

The 10 μL reaction mixture included 0.2 μL of 50 × ROX dye, 0.5 μL of each primer (10 pmol/μL), and nuclease-free water. Thermal cycling involved an initial denaturation at 95°C for 10 min, followed by 40 cycles of 95°C for 15 s and 58°C for 1 min. Gene expression levels of IL-6, IL-1β, IFN-γ, and caspase-3 were quantified with β-actin, which was used as a housekeeping gene for normalization.

The relative fold changes were determined using the 2^−ΔΔCt^ method. Each sample was analyzed in triplicate to ensure data reliability and accuracy. Detailed primer sequences and references are provided in Table 1.

**Table 1:** Primers used in the transcription levels of the proinflammatory cytokines.

Gene	Forward	Reverse	Accession number	References
Interleukin 1β	CTGCCTGCAGAAGAAACCCC	GGACGGTACAGAGCGATGTT	NM_001282824	(46)
Interleukin 6	AGCGTCGATTTGCTGTGCT	GATTCCTGGGTAGCTGGGTCT	AB618538.1	(47)
Caspase-3	GATGGCCCTCTTGAACTGAA	AGAGCTTGGGTTTTCCTGCT	AB618543	(47)
IFN-γ	CAAGTCAAAGGCGCACGTC	GCGTTGAGTTTTCAAGTCATTC	NM_001282845	(48)
β-actin	AGGCTACAGCTTCACCACCAC	CCATCTCCTGCTCAAAATCCA	AB980793.1	(49)

### Histopathological examination

The tissue specimens from the heart, lungs, liver, and spleen of the infected pigeon were fixed in 10% buffered neutral formalin and processed routinely and stained with hematoxylin and eosin (20).

The stained tissue sections were viewed, and the consistency of meronts was carefully examined using Olympus BH-2 microscope. The lesions and meronts were photographed using an Olympus DP27 camera linked to software (cellSens dimensions, version 1.13).

### Statistical analysis

Python (pandas, scipy, and seaborn) and GraphPad Prism were used for all statistical analyses. First, the Shapiro–Wilk and Levene’s tests were used to check the data for normality and homogeneity of variance, respectively. Contingency tables comparing infectivity status across categorical groups were subjected to a chi-square test of independence to assess the impact of age, sex, and location on infection prevalence.

Transcript levels of IL-6, IL-1β, IFN-γ, and caspase-3 were measured using the 2^−ΔΔCt^ method and normalized to β-actin for gene expression analysis. Independent samples t-tests were used to evaluate differences in gene expression between tissue types (lung versus spleen), with significance defined at *P*<0.05. Bar charts with mean values ± standard deviation (SD) and standard error bars were used to show gene expression data, and the plots were annotated with *P* values.

## Results

### Clinical signs and postmortem (PM) examination

**Figure 1:**
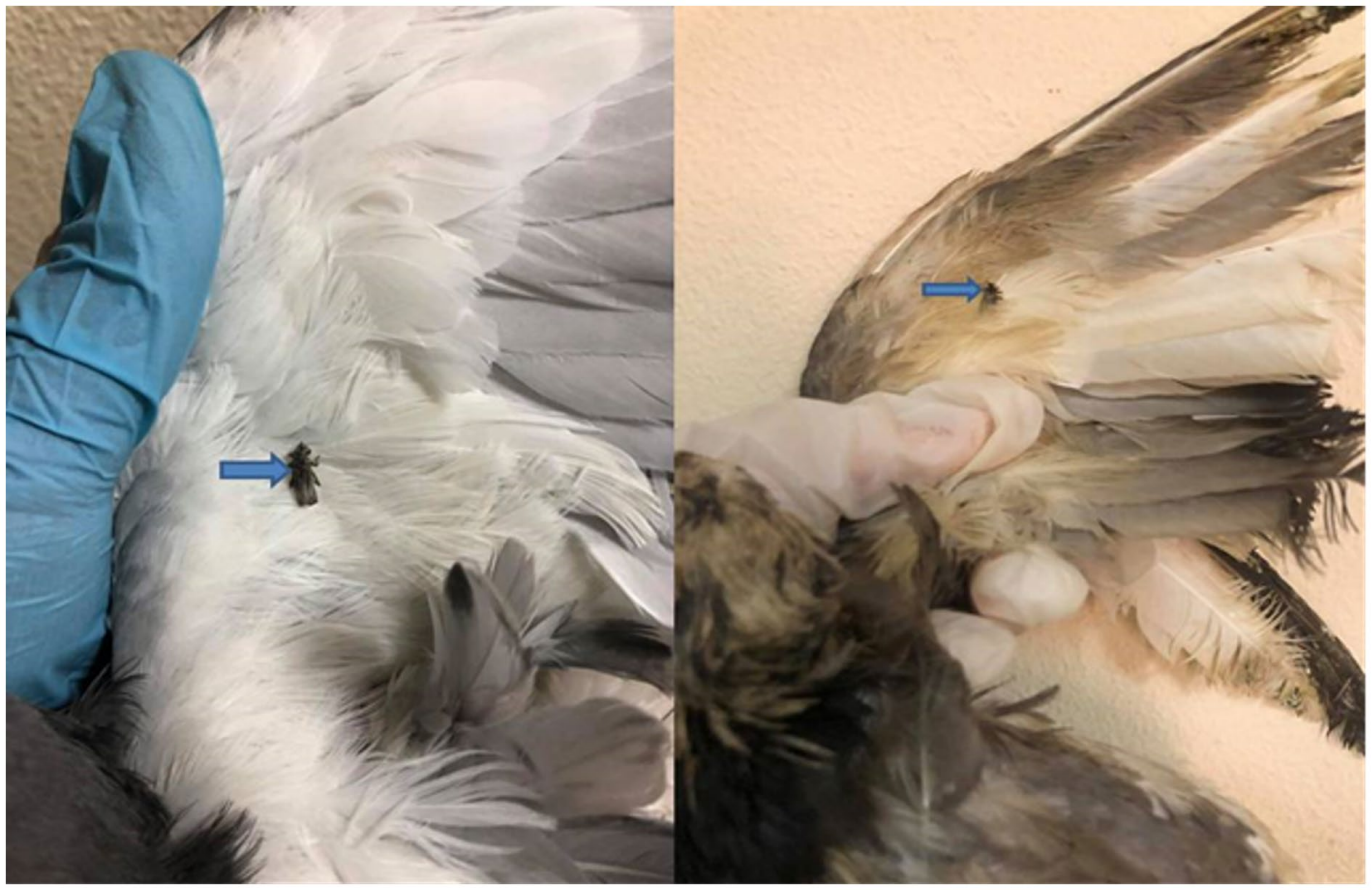
Pigeon infested with *Pseudolynchia canariensis* (the pigeon louse fly or pigeon fly).

Hippoboscid flies were identified when examining pigeon bodies. These flies were collected from nine infected birds, and upon closer inspection, they were identified as *P. canariensis*, a pigeon dipteran parasite (Figure 1).

**Figure 2:**
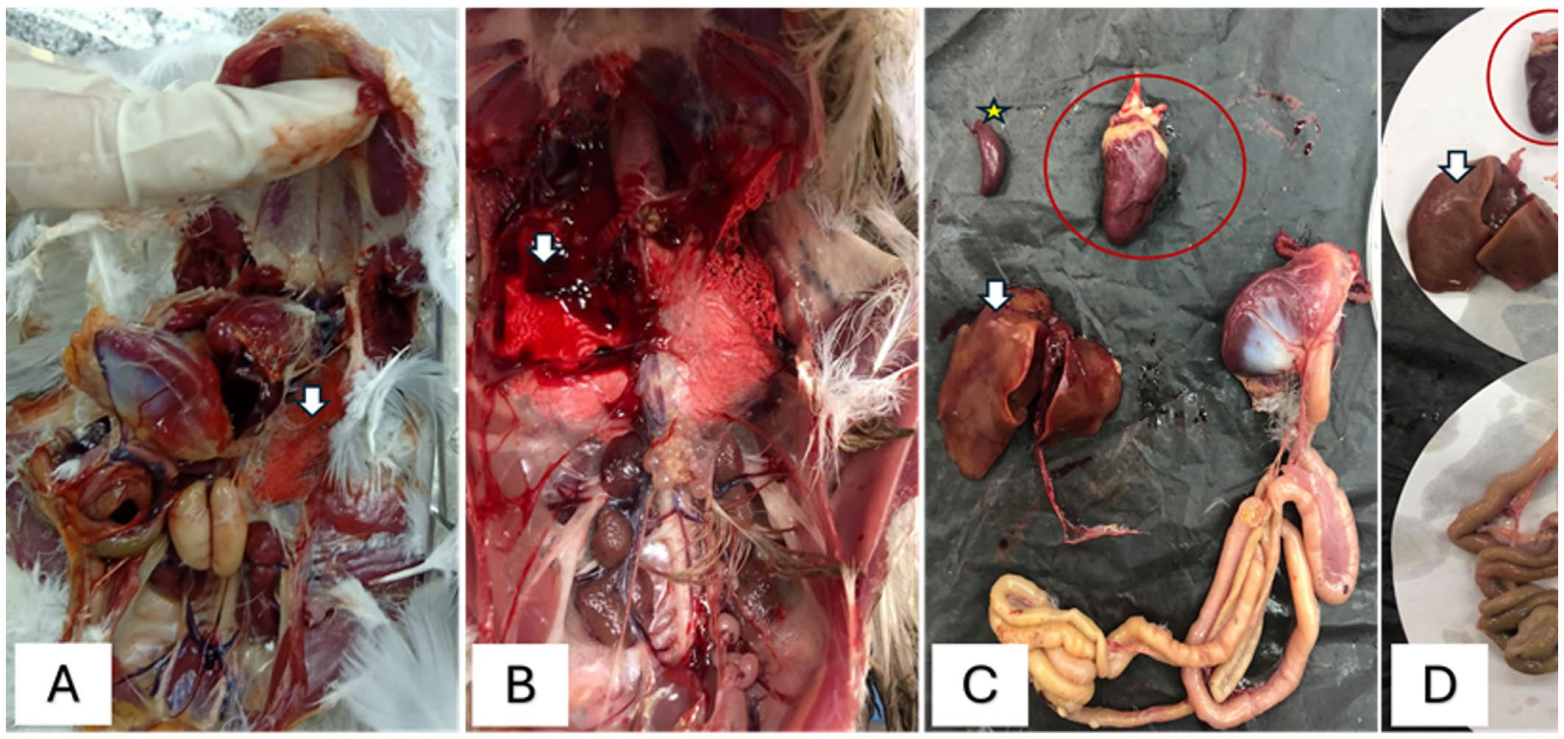
Postmortem lesion of freshly dead pigeons showing **(A)** congested lung (white arrow). **(B)** Focal pneumonia (white arrow). **(C)** Congested spleen (yellow star) and enlarged heart (red ring). **(D)** Pale and degenerated liver (white arrow) and dark, congested heart (red ring).

The examined birds exhibited general signs of depression, emaciation, anemic appearance, ruffled feathers, and some birds showed nervous and/or respiratory manifestations. The PM lesions had significant splenic congestion, with hemorrhages seen in the thigh and breast muscles, alongside cardiomegaly and enlargement, as well as congestion in the majority of parenchymatous organs. Pneumonia was also reported in some cases (Figure 2).

None of the variables—age, sex, or locality (region)—showed a statistically significant association with infectivity status (all *P* values were >0.05). This indicates that differences in infection rates across these groups may be due to random variation rather than meaningful demographic or geographic trends (Tables 2, 3).

**Table 2:** Infectivity in relation to age, sex, and locality.

Variable	Category	Negative	Positive
Age group	0–6 months	28	3
Age group	7–12 months	7	0
Age group	13–24 months	29	1
Sex	Female	23	3
Sex	Male	41	1
Region	Fayoum	29	1
Region	Giza	35	3

**Table 3:** Chi-square test results: significance of infectivity by group.

Variable	*p*-value	Interpretation
Age group	0.450	Not significant
Sex	0.303	Not significant
Region	0.784	Not significant

### Parasitological examination

**Figure 3:**
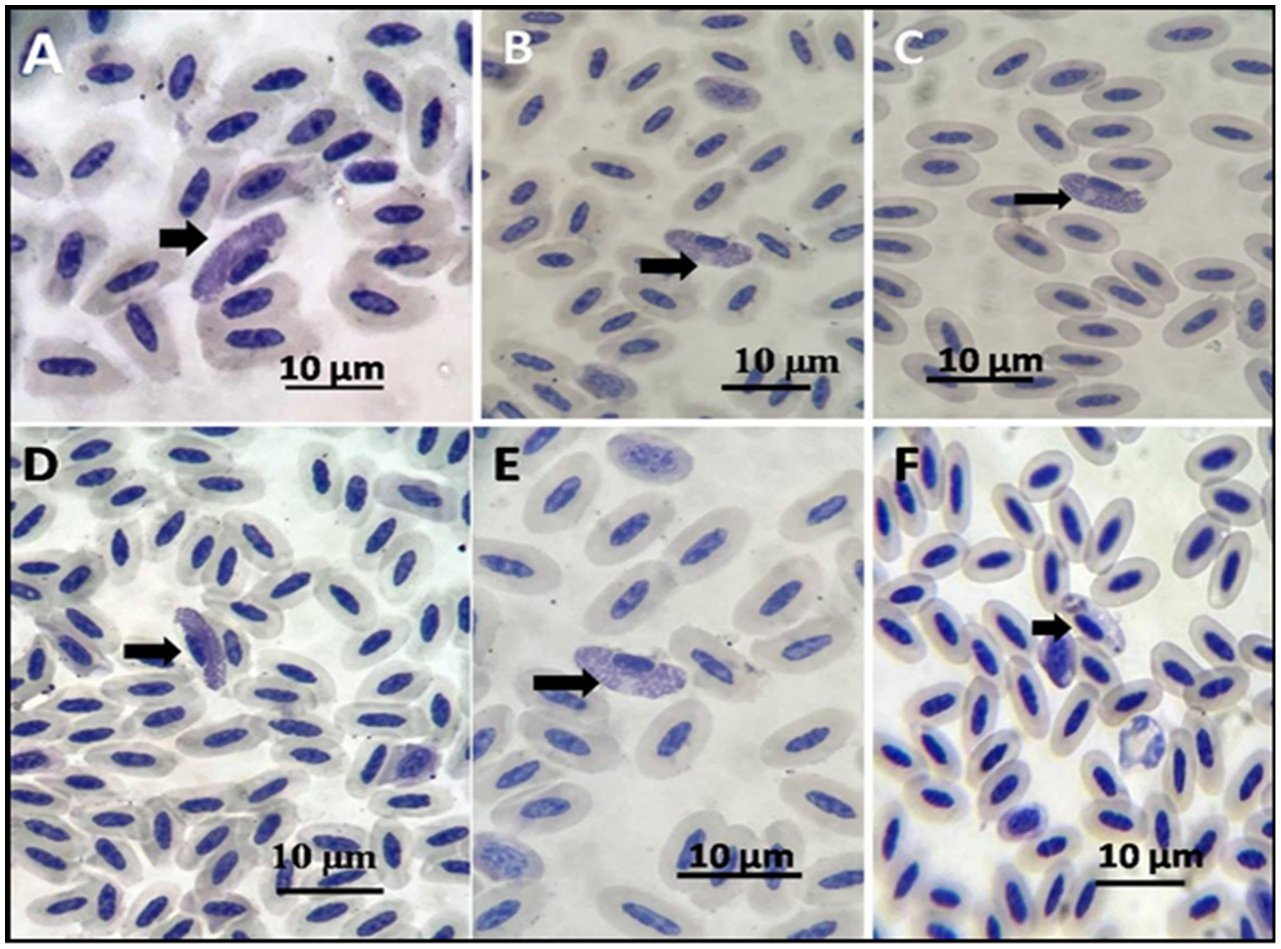
Pigeon-stained thin blood film with Giemsa stain showing **(A–E)** macrogametocytes shown by arrows. **(F)** Microgametocyte.

Examining every positive case revealed that the infected erythrocytes of the pigeons contained 1–2 gametocytes per 10 microscopic fields, with an incidence of 8% infected pigeons (10/125). The gametocytes encircled the host cell nucleus, either male or female. The male or microgametocyte had a light red cytoplasm with granules at two poles, while the macrogametocyte had a dark cytoplasm with diffused granules (Figure 3).

### Transcript levels of the cytokines in the infected spleen and lung of the pigeons

Gene expression analysis showed upregulation of IL-6, IL-1β, and IFN-γ in infected tissues. Mean values were higher in lungs than spleens, though not statistically significant (IL-6 *P* = 0.226; IL-1β *P* = 0.593; IFN-γ, *P* = 1.000).

**Figure 4:**
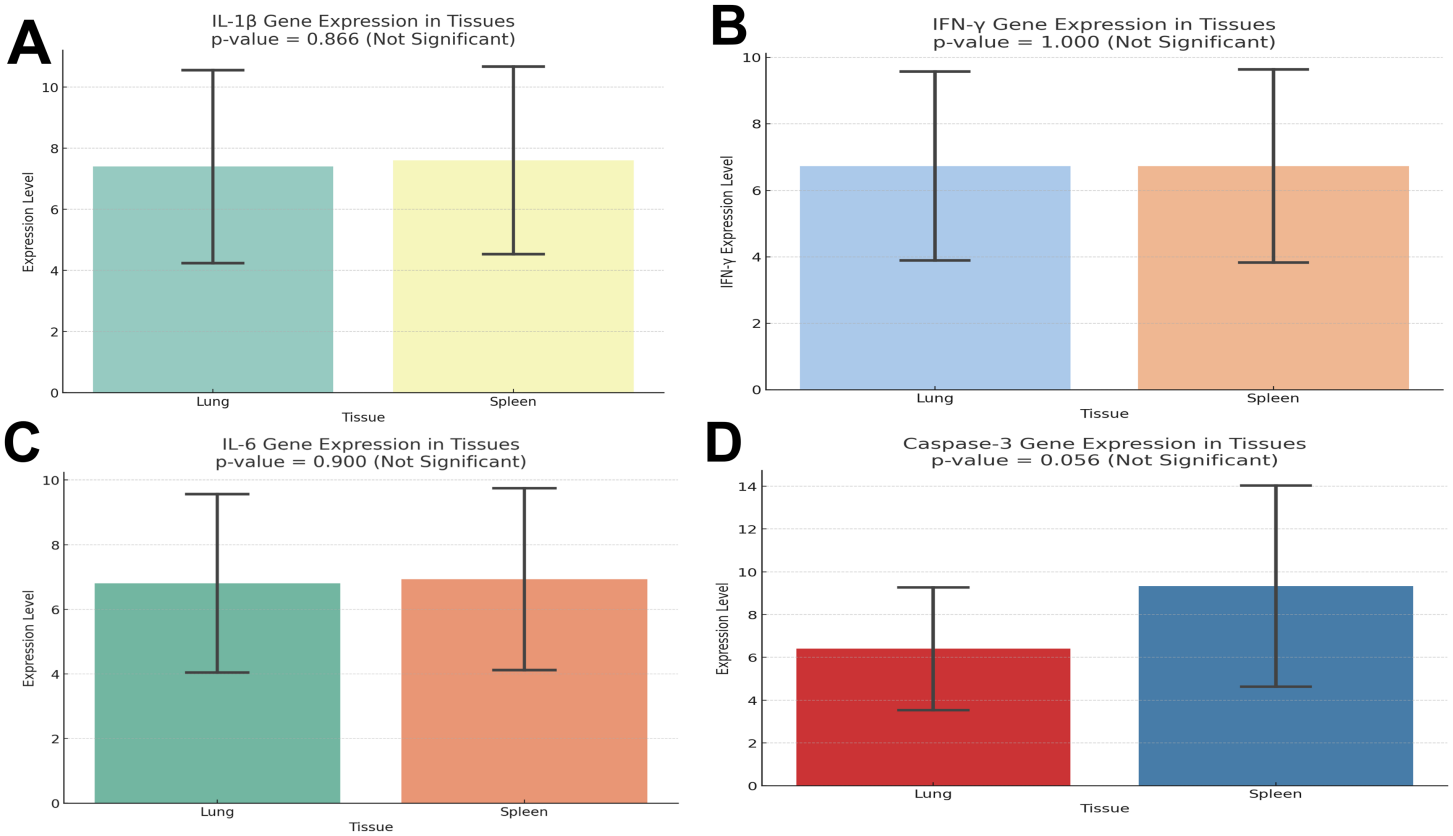
**(A)** Transcript levels of interleukin 1β in lung and spleen of the infected and control pigeon. **(B)** Transcript levels of IFN-γ in lung and spleen of the infected and control pigeon. **(C)** Transcript levels of IL-6 in lung and spleen of the infected and control pigeon. **(D)** Transcript levels of caspase-3 in lung and spleen of the infected and control pigeon.

Caspase-3 was also elevated, suggesting apoptosis (Figures 4A–D). Cytokine expression shows the mean ± SD of each marker (IL-6, IL-1β, IFN-γ, caspase-3) in lung and spleen tissues, with *P* values and significance interpretation (Table 4).

**Table 4:** Cytokine expression levels in lungs and spleen of the pigeons.

Marker	Mean expression (lung)	SD (lung)	Mean expression (spleen)	SD (spleen)	*p*-value	Significance
IL-6	6.8	2.86	6.93	2.91	0.226	Not significant
IL-1β	7.4	3.27	7.6	3.18	0.593	Not significant
IFN-γ	6.73	2.94	6.73	3.01	1.0	Not significant
Caspase-3	6.4	2.97	9.33	4.86	0.079	Not significant

### Histopathological findings

**Figure 5:**
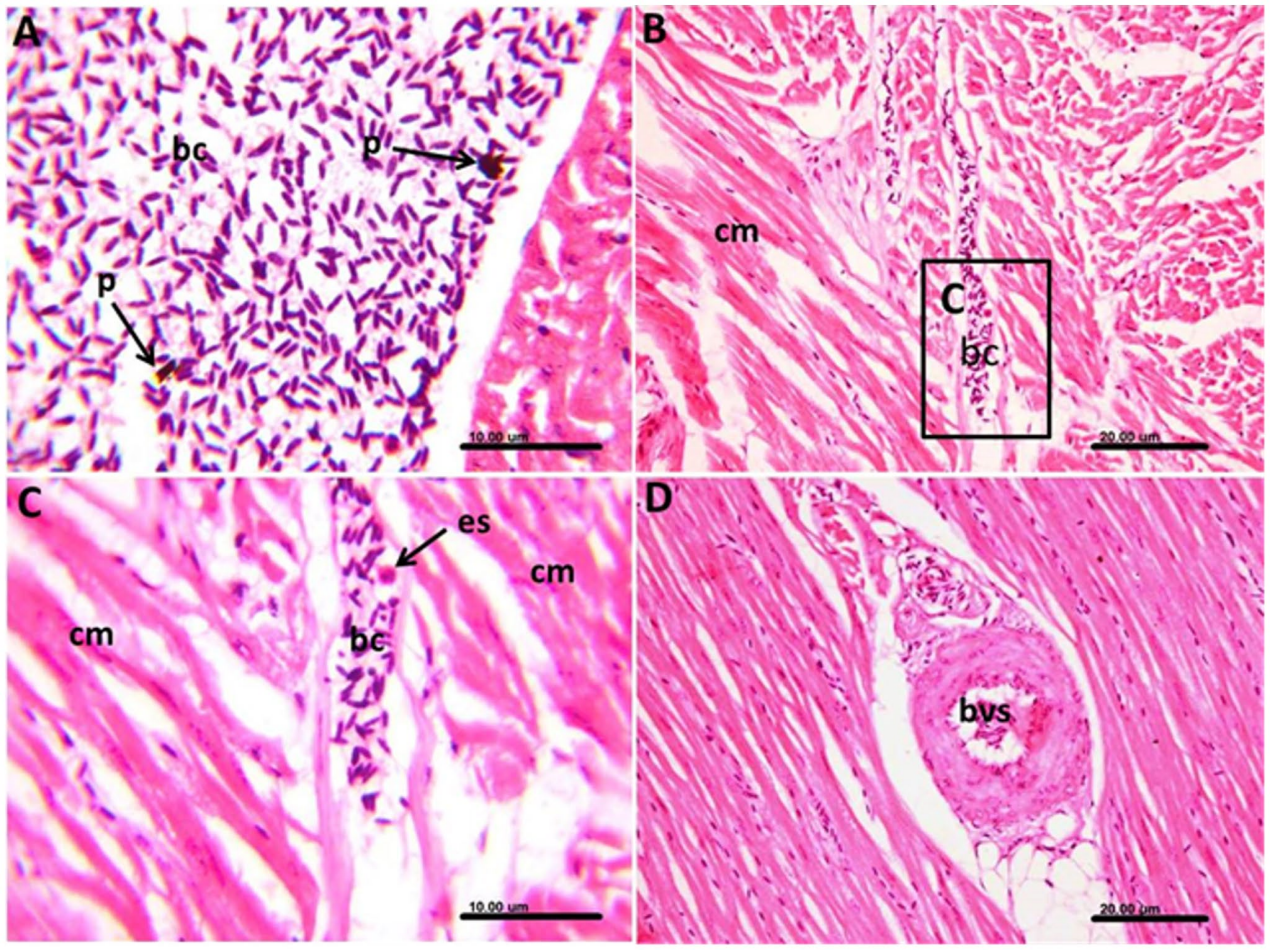
Photomicrograph of the heart of *Columba livia* infected with *Haemoproteus columbae* and stained with hematoxylin and eosin showing **(A)** infected blood cells (bc) with abnormal elongated shape with granular basophilic cytoplasm; these cells contain the stages of hemoproteus parasite (p). **(B)** The infected cells are observed between the cardiac muscles (cm) with edema and hyalinosis of the muscle fibers. **(C)** Higher magnification of the previous image showing the infected blood cells (bc) containing hemoproteus stages within the blood capillary together with eosinophils (es). **(D)** Blood vessel (bvs) in the cardiac muscle showing swelling of the endothelium and thickening of its wall.

The lesions in the infected pigeon were very clear in internal organs, including the heart, lungs, liver, and spleen (Table 5). The lesions were distinct in the blood vessels of such organs and were associated with degenerative and inflammatory reactions. In the heart, the infected blood cells within the blood vessels appeared abnormally elongated in shape. The cells contained granular, elongated basophilic stages of *Haemoproteus* in the cytoplasm, representing the developing stages of this parasite (Figure 5A).

**Table 5:** Lesions in different organs of the pigeons.

Tissue	Lesion description
Spleen	Arteriolar wall thickening, white pulp activation
Liver	Lymphocytic perivascular infiltration, hepatocyte degeneration
Heart	Parasite in vessels, edema, and myocyte degeneration
Lung	Congestion, inflammatory, homozoin pigments

The infected blood cells were also noticed in the deep blood capillaries, between the cardiac muscles. In such cases, the cardiac muscles showed edema and hyalinosis, along with a few eosinophil infiltrations (Figures 5B,C). The blood vessel itself showed swelling of the endothelium and thickening of its wall (Figure 5D).

**Figure 6:**
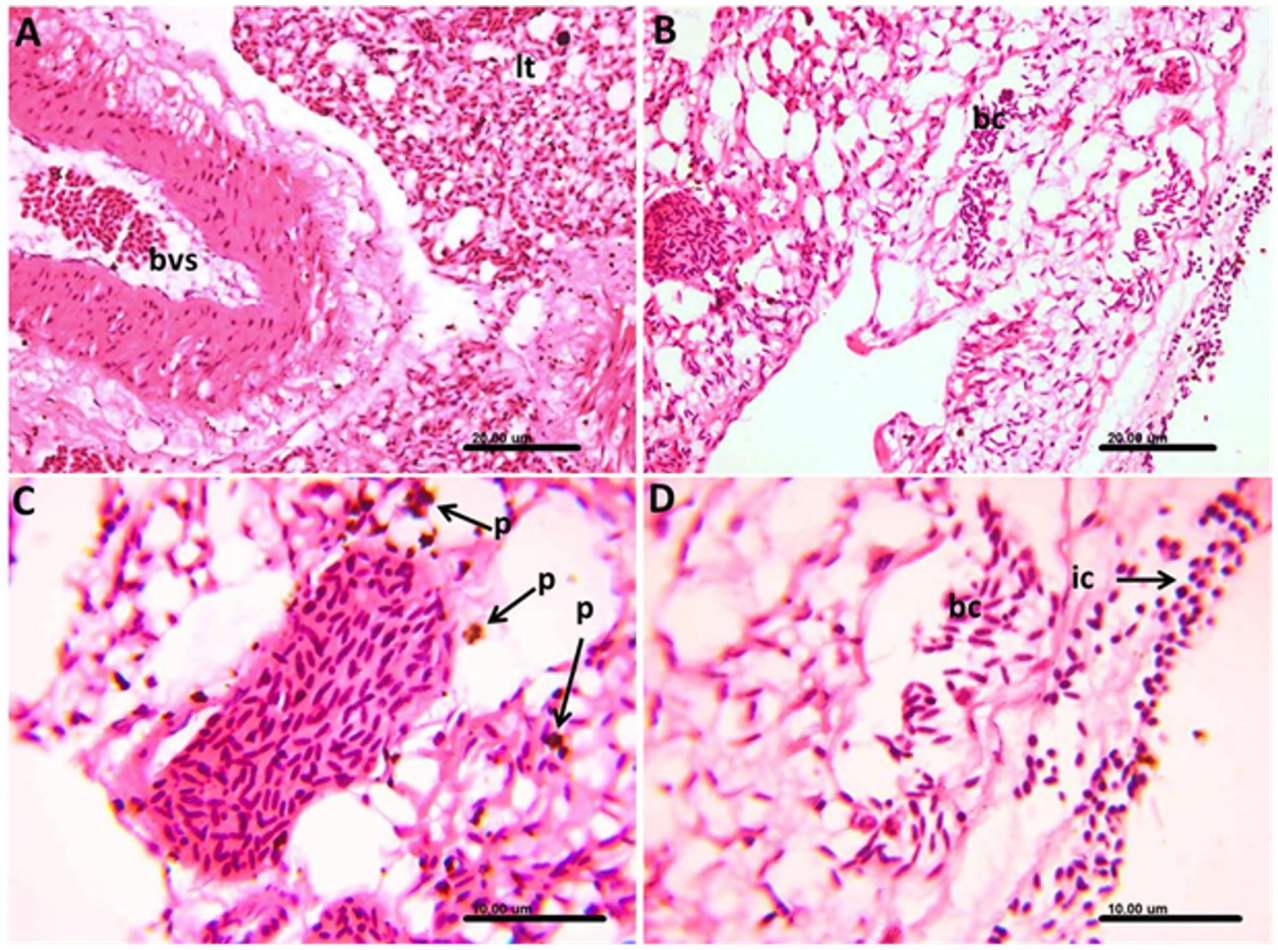
Photomicrograph of the lung of *Columba livia* infected with *Haemoproteus columbae*, and stained with hematoxylin and eosin showing **(A)** apparently normal nucleated red blood cells within the blood vessels (bvs) and congestion of the perialveolar capillaries of the lung tissue (lt). **(B)** Lung tissue showing infected blood cells (bc) containing the stages of hemoproteus parasite. **(C)** Infected blood cells surrounded by numerous hemozoin pigments (p). **(D)** Infected blood cells (bc) in the lung tissue surrounded by mononuclear inflammatory cells (ic) infiltration mainly lymphocytes and heterophils.

In the lung tissue, some parts of the lung tissue showed congestion of both large blood vessels and perialveolar capillaries and contained apparently normal nucleated red blood cells without stages of the *Haemoproteus* (Figure 6A). Other parts of the examined lung tissue showed infected blood cells containing the stages of *Haemoproteus* (Figure 6B). The infected cells in such cases were surrounded by numerous hemozoin pigments (Figure 6C). Mononuclear inflammatory cells infiltration, mainly lymphocytes and heterophils, was not uncommon (Figure 6D).

**Figure 7:**
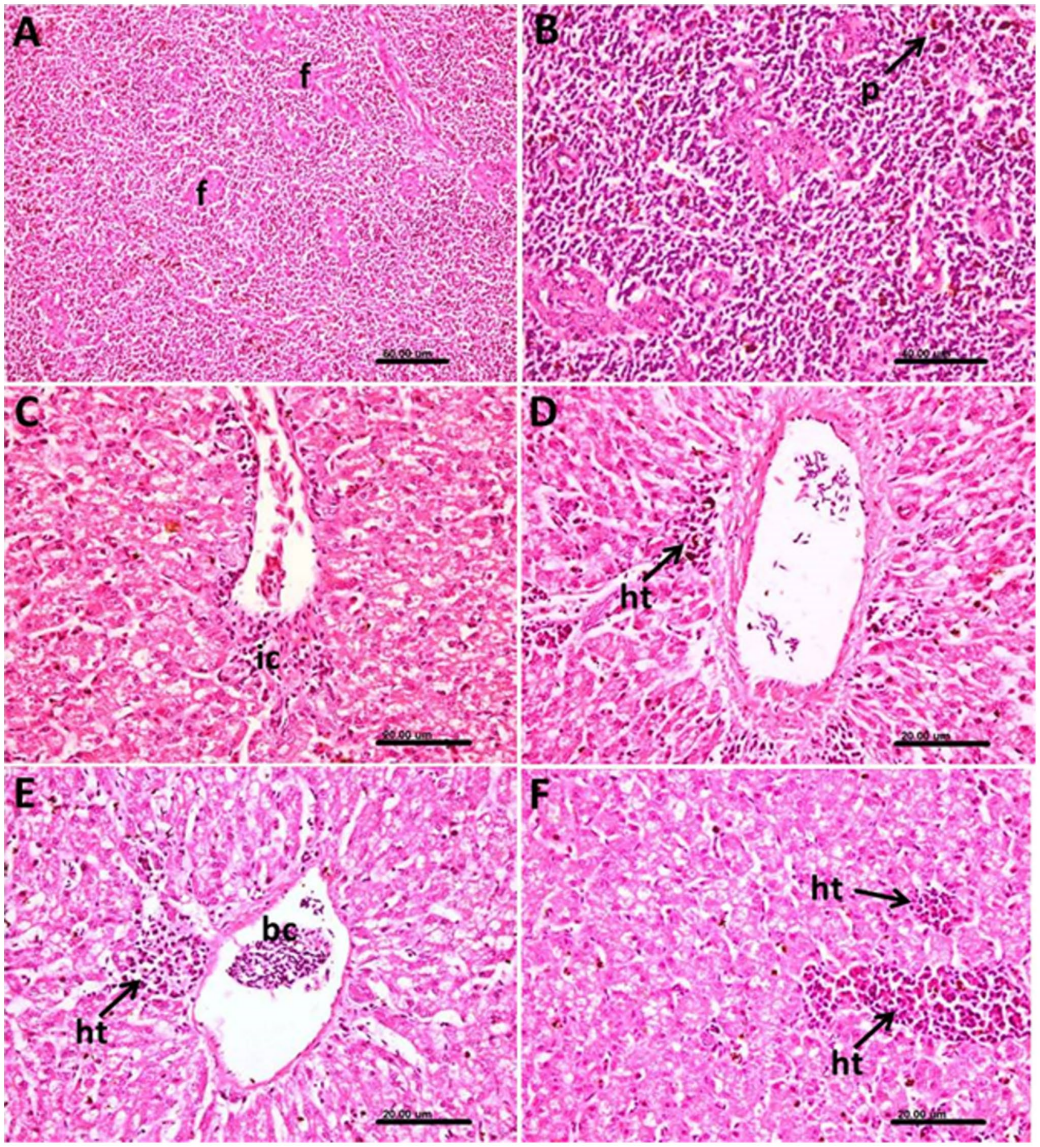
Photomicrograph of the spleen and liver of *Columba livia* infected with *Haemoproteus columbae* and stained with hematoxylin and eosin showing **(A)** thickening of the wall of the follicular arterioles (f) of the spleen. **(B)** Hemozoin pigments (p) in the splenic tissue. **(C)** Hepatic tissue showing mononuclear inflammatory cells (ic) infiltration mainly lymphocytes around the central vein. **(D)** Liver showing infected blood cells in the central vein and heterophils (ht) aggregation in the hepatic tissue. **(E)** Focal aggregation of heterophils (ht) and lymphocytes around the central vein containing infected blood cells (bc) and vacuolar degeneration of the hepatocytes. **(F)** Multiple foci of heterophils (ht) and lymphocytes aggregation in the hepatic tissues.

In the spleen, marked thickening in the wall of follicular arterioles was common, and the white pulp showed activation of the hemopoietic tissue together with hemozoin pigment deposition (Figures 7A,B).

In liver, the pathological changes were prominent where perivascular mononuclear inflammatory cells infiltration mainly lymphocytes around the central veins were common (Figure 7C), and the infected blood cells within the central vein were also noticed (Figure 7D). In such cases, a heterophils aggregation was also observed.

The heterophils and lymphocytes were sometimes noticed as a focal aggregation around the central vein containing infected blood cells (Figure 7E) or as multiple foci aggregated extravascular in the hepatic tissue (Figure 7F). The hepatocytes in such cases showed vacuolar degeneration.

## Discussion

*Haemoproteus* infections are common in wild birds; however, they are not usually deadly (21, 22). Nonetheless, Hartley (23) reported deaths in Australian wild currawongs (*Strepera graculind*). The study’s high infection was largely caused by the louse fly, *P. canariensis*, a known vector of *H. columbae* (24, 25). This was probably the outcome of repeated re-infection with *H. columbae* in captivity. In Saudi Arabia, domestic pigeons were found to harbor *H. columbae* (26).

However, in the current study, a macroscopic and histological investigation showed a correlation between the tissue stages of *H. columbae* megaloschizonts and the cardiac muscle lesions. The widespread necrotic lesions in the legs, wings, and breast muscles resembled lesions caused by nutritional myopathy resulting from a selenium or vitamin E deficiency. Comparable results have been recorded in many studies by Simpson (27), Atkinson et al. (28), Gardiner et al. (29), and Atkinson et al. (30).

According to Cepeda et al. (31), the pigeon used in this investigation contained thin-walled aseptate elongated, spherical, or irregularly shaped schizonts within the lumina of blood arteries in the lung, liver, kidney, and proventriculus. According to Kucera et al. (32), these schizonts contained merozoites, cytomeres, or other forms. It has been documented that birds infected during the prepatent period perish have severe hemoproteosis as a result of the rupture of megalomeronts in the muscles and liver and the subsequent pathogenic changes as suggested by Youssefi et al. (33).

Natural *Haemoproteus* infection and subsequent consequences analysis have demonstrated that haemoproteosis can result in a remarkable pressure on the hosts’ survival, behavior, reproductive efficiency and community framework (34, 35). In feral domestic pigeons living, the amount of *H. columbae* parasitemia and body mass are negatively correlated. In the present investigation, these could lead to body mass loss and other adverse health outcomes that in consistent with Youssefi et al. (33), and Nebel et al. (36).

A significant disease was brought on by the pigeon’s increased gametocyte to erythrocyte ratio. The infected erythrocyte counts inside the microscopic field in the positive instances, which demonstrated greater affection of 1–2 erythrocytes, also indicative of milder infection in the birds, supported the findings of Mushi et al. (37), and Gicik and Arslan (38).

It is well accepted that the pathogenicity of *Haemoproteus* spp. in their natural avian hosts is essentially negligible, particularly in comparison to the pathology of the avian hosts of the genera *Plasmodium* and *Leucocytozoon*. Inadvertent hosts, like captive avian species in zoos and aviaries, have been shown to get clinical and lethal *Haemoproteus* infections (39, 40). In this regard, schizonts were detected in the current study at the lungs’ vascular periphery, indicating development among endothelial cells. Despite the study’s lack of finding any correlation between schizonts and endothelial cells, it was not always possible to pinpoint the precise kind and developmental stage of the structures inside the megaschizonts (37).

The host’s defenses against infection by foreign microorganisms depend on a functioning immune response. Intense reactions could be harmful to the host. In our study, the transcript levels of inflammatory markers such as IL-6, IFN-γ, and IL-1β were found to be significantly upregulated in *H. columbae* infected birds. Cytokines play an important role and are considered as an effective element of the avian immunity (41–43).

They are usually considered in case of parasitic infections, especially intracellular parasites (44). On the other hand, and in contrary to endoerythrocytic parasites, Mohammed (24) and Rennenberg et al. (45) have paid attention to the effect of cysteine protease inhibitor, which is capable of blocking cell death (apoptosis) of host hepatocytes in the case of exoerythrocytic *Plasmodium* infection. However, the endoerythrocytic nature of *Hemoproteus* infection could explain the increased apoptotic activity marker (Cas-3) in our study.

IFN-γ is a vital Th1 cytokine that promotes antiviral immunity and activates macrophages. There was no tissue-specific immune activation, as indicated by the equal expression in the spleen and lung. This could suggest a homeostatic state in which both organs’ baseline levels of IFN-γ are maintained. Additionally, the lack of differential expression suggests that there was no active inflammation or infection in the tissues collected at the time of assessment.

Fever induction and early innate immune responses are significantly influenced by IL-1β. Its comparatively consistent expression in different tissues points to widespread surveillance activity in the spleen and lung, which may be connected to the lymphoid and mucosal compartments’ mutual requirement for cytokine responsiveness. A multipurpose cytokine, IL-6 plays a role in immunological responses and inflammation. Its similar expression in the two tissues would indicate a baseline level of IL-6 activity across the body, most likely in a non-inflammatory or steady-state state. This implies that, in the circumstances examined, neither tissue exhibits preferential IL-6 upregulation.

The spleen of infected pigeons had higher transcriptional activity (mean = 9.33 ± 4.86) than the lung (mean = 6.40 ± 2.97), according to the gene expression study of caspase-3, a crucial apoptotic marker. The difference did not achieve statistical significance (*P* = 0.079), despite the fact that this trend suggests increased apoptotic signaling in splenic tissue. These findings imply that although there is an increase in caspase-3 expression, which might be due to tissue-specific immune responses or parasite-induced apoptosis, the ability to detect a statistically significant difference may have been diminished by the variability among biological replicates or the small sample size. However, the observed rise is consistent with the histologically documented tissue damage and inflammatory profile, hence corroborating the significance of apoptosis in the pathophysiology of *H. columbae* infection.

Although *H. columbae* typically causes subclinical infections, this study revealed histological lesions in vital organs and mild immune activation. Cytokine transcription was elevated but not statistically significant, possibly due to the small sample size (*n* = 5 per group). These results mirror findings in accidental hosts where immune dysregulation and pathology are more severe.

Our qPCR data align with prior studies on avian cytokine response during parasitic infections (41–43). The lack of statistical significance may reflect limitations in power and inter-individual variability. Histopathologically, the presence of parasites in endothelial and parenchymal tissues suggests developmental schizogony stages. These lesions, along with cytokine upregulation and caspase-3 activity, underscore the potential for immune-mediated tissue damage.

The circular basophilic globular aggregates resemble cytomeres, as reported by Gardiner et al. (29) and Msoffe et al. (39). There are a few incomplete septa that resemble the outer walls, which suggest that the megaloschizont’s compartmentalization was caused by ingrowth from the walls. The morphology of both doves’ merozoites and the descriptions of merozoites from other *Haemoproteus* species showed a favorable correlation (28, 30).

## Conclusion

In the current study, *H. columbae* was detected in domesticated farmed pigeons with an incidence of 8%. However, *H. columbae* causes a low mortality rate, and the infected birds suffered from severe pathological changes in most hematopoietic and parenchymatous organs. The transcript levels of inflammatory markers such as IL-6, IFN-γ, and IL-1β were found to be significantly upregulated in *H. columbae* infected birds. Additionally, the mRNA level of the apoptotic Cas-3 in the *H. columbae* samples indicated apoptotic activity. Further studies are recommended to study the effect of different herbal extracts in comparison to the most commonly used drugs in controlling such a problem in infected pigeons.

## Data Availability

The raw data supporting the conclusions of this article will be made available by the authors, without undue reservation.
